# Efficacy and Safety of Endoscopic Ultrasound‐Guided Biliary Drainage in the Management of Malignant Hilar Biliary Obstruction: A Multicenter Retrospective Cohort Study

**DOI:** 10.1002/deo2.70413

**Published:** 2026-08-24

**Authors:** Takuji Iwashita, Kazuki Hayashi, Michihiro Yoshida, Reiko Yamada, Koichiro Matsuda, Shinichi Morita, Tadahisa Inoue, Kazuhiko Shioji, Keisuke Iwata, Akinori Maruta, Nobuhiko Hayashi, Masaaki Yano, Ichiro Yasuda

**Affiliations:** ^1^ Department of Gastroenterology Shiga University of Medical Science Shiga Japan; ^2^ First Department of Internal Medicine Gifu University Hospital Gifu Japan; ^3^ Department of Gastroenterology Nagoya City University East Medical Center Aichi Japan; ^4^ Department of Gastroenterology and Metabolism Nagoya City University Graduate School of Medical Sciences Aichi Japan; ^5^ Department of Gastroenterology and Hepatology Mie University Graduate School of Medicine Mie Japan; ^6^ Department of Gastroenterology Toyama Prefectural Central Hospital Toyama Japan; ^7^ Department of Gastroenterology and Hepatology Niigata Prefectural Central Hospital Niigata Japan; ^8^ Department of Gastroenterology Aichi Medical University Aichi Japan; ^9^ Department of Gastroenterology Niigata Cancer Center Hospital Niigata Japan; ^10^ Department of Gastroenterology Gifu Municipal Hospital Gifu Japan; ^11^ Department of Gastroenterology Gifu Prefectural General Medical Center Gifu Japan; ^12^ Third Department of Internal Medicine University of Toyama Toyama Japan; ^13^ Department of Gastroenterology Kurobe City Hospital Toyama Japan

**Keywords:** antegrade stenting, bridging stenting, choledochoduodenostomy, endoscopic retrograde cholangiopancreatography (ERCP), hepaticogastrostomy (HGS)

## Abstract

Malignant hilar biliary obstruction (MHBO) remains challenging for biliary decompression. Although endoscopic ultrasound‐guided biliary drainage (EUS‐BD) has emerged as an alternative to endoscopic retrograde cholangiopancreatography‐guided BD (ERCP‐BD), evidence regarding EUS‐BD for MHBO is limited.

**Aims:**

To evaluate the efficacy and safety of EUS‐BD for MHBO.

**Methods:**

This multicenter retrospective cohort study included patients with MHBO who underwent EUS‐BD at 12 tertiary referral centers between 2012 and 2023. Technical success, clinical success, and procedure‐related adverse events (AEs) were assessed. Multivariable analysis was performed to identify factors associated with clinical failure.

**Results:**

A total of 141 patients were included. EUS‐BD was performed as primary drainage in 67 patients (47.5%) and as rescue drainage after prior ERCP‐BD in 74 patients (52.5%); in rescue cases, the reported configurations represented only the EUS‐BD component. The overall technical success rate was 98.5% (139/141). Clinical success was achieved in 87.1% (121/139). Among patients who achieved clinical success, the median time to recurrent biliary obstruction was 201 days. Procedure‐related AE occurred in 15 patients (10.6%). Multivariable analysis identified Bismuth type IV strictures (odds ratio [OR], 5.89; 95% confidence interval [95% CI], 1.72–24.3) and EUS‐BD drainage limited to one or two liver segments (OR, 5.11; 95% CI, 1.49–21.2) as factors associated with clinical failure.

**Conclusions:**

EUS‐BD is a highly feasible and relatively safe option for BD of MHBO in both primary and rescue settings. Bismuth type IV strictures and EUS‐BD drainage limited to one or two liver segments were associated with clinical failure.

**Trial Registration:**

N/A

**Abbreviations**
AE: adverse event
AG: antegrade stenting
CI: confidence interval
ERCP: endoscopic retrograde cholangiopancreatography
ERCP‐BD: endoscopic retrograde cholangiopancreatography‐guided biliary drainage
EUS: endoscopic ultrasound
EUS‐BD: endoscopic ultrasound‐guided biliary drainage
HDS: hepaticoduodenostomy
HGS: hepaticogastrostomy
HJS: hepaticojejunostomy
IHBD: intrahepatic bile duct
MDBO: malignant distal biliary obstruction
MHBO: malignant hilar biliary obstruction
OR: odds ratio
PS: plastic stent
PTBD: percutaneous transhepatic biliary drainage
SEMS: self‐expandable metallic stent
T‐Bil: total bilirubin

## 1 Background

Malignant hilar biliary obstruction (MHBO) is technically challenging owing to the complex biliary anatomy. Precise delineation of the biliary anatomy, obstructed segments, and determination of the optimal drainage area is essential for achieving effective biliary decompression in the management of MHBO [1]. Adequate drainage is crucial for relieving symptoms and improving hepatic function to enable subsequent anticancer therapy, thereby enhancing both prognosis and quality of life.

Percutaneous transhepatic biliary drainage (PTBD) or endoscopic transpapillary biliary drainage using the endoscopic retrograde cholangiopancreatography (ERCP‐BD) has been recommended for biliary decompression in patients with unresectable MHBO [2]. ERCP‐BD is generally preferred because it avoids percutaneous access and external drainage. However, ERCP‐BD is technically more challenging in MHBO than in malignant distal biliary obstruction (MDBO), particularly in patients with advanced Bismuth type III or IV strictures or complete ductal occlusion [3, 4].

Endoscopic ultrasound‐guided BD (EUS‐BD) has recently emerged as an alternative or rescue technique when ERCP‐BD is unsuccessful or not feasible [5, 6]. EUS‐BD enables direct access to the bile ducts through the gastric or duodenal wall and allows various drainage strategies, including hepaticogastrostomy (HGS), choledochoduodenostomy, and antegrade stenting (AG) [5, 7, 8, 9]. While EUS‐BD has been increasingly adopted for MDBO [6, 7], evidence regarding its specific role in the management of MHBO remains limited. Therefore, the present study aimed to evaluate the technical success, clinical success, adverse events (AEs), and factors associated with clinical failure of EUS‐BD in patients with MHBO using a large multicenter retrospective cohort.

## 2 Patients and Methods

### 2.1 Study Design

This multicenter retrospective cohort study was conducted across 12 tertiary referral centers (institution numbers 2–13) with expertise in advanced pancreatobiliary endoscopy. A retrospective database analysis of all patients who underwent EUS‐BD between April 2012 and March 2023 was performed to identify eligible patients according to the following inclusion and exclusion criteria. The inclusion criteria were as follows: (1) patients who underwent EUS‐BD for unresectable MHBO; (2) patients with MHBO diagnosed based on imaging studies and/or histopathological findings; and (3) patients aged ≥18 years. Patients who underwent EUS‐BD for benign biliary strictures or MDBO were excluded. This study was conducted in accordance with the Declaration of Helsinki. The study protocol was approved by the Institutional Review Board of Gifu University Hospital (GUH; approval number 2024‐019), which served as the central ethics committee covering all participating institutions.

### 2.2 EUS‐BD Procedures

EUS‐BD was performed using a convex‐type echoendoscope. After visualization of the intrahepatic bile duct (IHBD) from the stomach or duodenum, the bile duct was punctured under EUS guidance using a 19‐ or 22‐gauge fine‐needle aspiration needle. After cholangiography, a guidewire was inserted, the tract was dilated, and a plastic stent (PS) or self‐expandable metallic stent (SEMS) was placed according to the planned drainage route. HGS or hepaticoduodenostomy (HDS) was performed using a PS or partially covered SEMS, whereas bridging stenting or AG was performed using an uncovered SEMS. The selection of drainage modality and EUS‐BD technique was determined at the discretion of the endoscopist at each participating institution. Primary EUS‐BD was selected after considering multiple factors, including biliary anatomy, the location and extent of hilar obstruction, the anticipated difficulty of ERCP‐BD, gastrointestinal anatomy, the presence of intestinal obstruction or cholangitis, and the patient's general condition. Primary EUS‐BD was not selected according to a standardized protocol and was not intended to represent a universally recommended first‐line strategy for MHBO. The EUS‐BD technique and stent configuration were selected at the discretion of the individual endoscopist at each participating institution. The number of drained liver segments was defined as the number of segments drained by EUS‐BD alone and did not include segments drained by previously placed transpapillary stents.

### 2.3 Study Definitions

Primary (first‐line) EUS‐BD was defined as EUS‐BD selected as the initial BD modality. Rescue EUS‐BD was defined as EUS‐BD performed after prior ERCP‐based BD. This included patients in whom ERCP‐BD was technically unsuccessful and those in whom ERCP‐BD was technically successful but provided insufficient drainage because one or more obstructed biliary segments remained undrained. All EUS‐BD procedures, including combined stent configurations, were performed during a single endoscopic session. Technical success was defined as successful completion of the intended stent configuration with appropriate stent placement for the target bile ducts. Clinical success was defined as improvement in biliary obstruction‐related symptoms or, in patients with obstructive jaundice, a reduction in total serum bilirubin (T‐Bil) of ≥ 50% within 14 days after the procedure. In patients who had undergone prior ERCP‐BD, clinical success was assessed based on the residual symptoms, cholangitis, or T‐Bil level immediately before EUS‐BD. AEs were recorded and graded according to a lexicon for endoscopic AEs: report of the American Society for Gastrointestinal Endoscopy workshop [10]. Time to recurrent biliary obstruction (TRBO) was evaluated in the patients who achieved clinical success after technically successful EUS‐BD. RBO was defined as the recurrence of biliary obstruction‐related symptoms, cholangitis, or biochemical evidence of biliary obstruction requiring additional biliary intervention after initial clinical success. TRBO was calculated from the date of EUS‐BD to the date of recurrent biliary obstruction. Patients without recurrent biliary obstruction were censored at the date of death or the last follow‐up.

### 2.4 Study Outcomes and Statistical Analysis

The study outcomes were technical success, clinical success, TRBO, procedure‐related AE, and factors associated with clinical failure. Continuous variables are presented as the median and range. Technical success, clinical success, and AE rates were calculated with 95% confidence intervals (CIs). Statistical comparisons were performed using Fisher's exact test for categorical variables and the Mann–Whitney U test for continuous variables. Potential factors associated with clinical failure of EUS‐BD were evaluated using univariable and multivariable logistic regression analyses. Candidate variables were selected a priori based on clinical relevance and potential association with clinical failure in EUS‐BD for MHBO. These variables included age, sex, Bismuth type IV obstruction, primary versus rescue EUS‐BD, and EUS‐BD drainage limited to one or two liver segments. Because the number of clinical failure events was limited, the multivariable analysis was considered exploratory. A two‐sided *p*‐value <0.05 was considered statistically significant. TRBO was estimated using the Kaplan–Meier method. The median TRBO and the corresponding 95% CI were calculated. All statistical analyses were performed using JMP Student Edition version 18 (SAS Institute, Inc., Cary, NC, USA).

## 3 Results

### 3.1 Patient Characteristics

A total of 141 patients were included. The median age was 77 years, and 79 patients (56.0%) were male. Most patients had normal gastrointestinal anatomy (85.1%). The most common etiologies were hilar cholangiocarcinoma (27.7%), pancreatic cancer (20.6%), and gallbladder cancer (18.4%). Bismuth type IV obstruction was present in 51 patients (36.2%) (Table 1).

**TABLE 1:** Basic characteristics.

Overall patients	141 patients	
Age, median (range)		77 (33–94)
Gender, *n* (%)	Male	79 (56.0)
Anatomy, *n* (%)	Normal	120 (85.1)
	RY	6 (4.3)
	PD	10 (7.1)
	Others	5 (3.5)
Etiology, *n* (%)	Hilar BDC	39 (27.7)
	Pancreatic cancer	29 (20.6)
	GB cancer	26 (18.4)
	Distal BDC	9 (6.4)
	Others	38 (27.0)
Bismuth type, *n* (%)	II	32 (22.7)
	IIIa	45 (31.9)
	IIIb	13 (9.2)
	IV	51 (36.2)

Abbreviations: BDC, bile duct cancer; GB, gallbladder; PD, pancreatoduodenectomy; RY, Roux‐en‐Y gastrectomy.

### 3.2 Details of EUS‐BD Procedures

**FIGURE 1:**
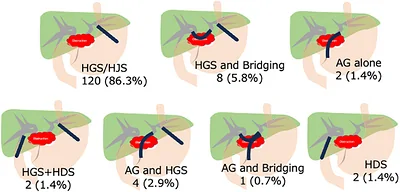
EUS‐BD stent configurations for MHBO. The configurations shown represent only the EUS‐BD component and do not include biliary drainage achieved by prior ERCP‐BD in patients undergoing rescue EUS‐BD. EUS, endoscopic ultrasound; BD, biliary drainage; MHBO, malignant hilar biliary obstruction; ERCP, endoscopic retrograde cholangiopancreatography; HGS, hepaticogastrostomy; AG, antegrade stenting; HDS, hepaticoduodenostomy.

EUS‐BD was performed as the primary BD modality in 67 patients (47.5%). In the remaining 74 patients (52.5%), EUS‐BD was performed as rescue drainage after prior ERCP‐BD and therefore constituted a combined ERCP‐BD and EUS‐BD approach. Indications for choosing EUS‐BD included inadequate or unsuccessful ERCP‐BD in 74 patients (52.5%), intestinal obstruction in 53 patients (37.6%), cholangitis in eight patients (5.7%), and surgically altered anatomy in six patients (4.3%). A 19‐gauge needle was used in the majority of procedures (85.8%), while a 22‐gauge needle was used in 14.2% of cases. The stent configurations described below represent only the EUS‐BD component and do not include stents previously placed by ERCP‐BD. In terms of EUS‐BD stent configuration, HGS, including hepaticojejunostomy (HJS), without additional EUS‐BD routes was the most frequently employed technique (86.3%). Combined approaches were also used, including HGS plus bridging (5.8%), HGS plus AG (2.9%), HGS plus HDS (1.4%), and other configurations. (Figure 1) The number of drained liver segments by EUS‐BD varied, with drainage of three segments being the most common (46.0%), followed by two segments (34.5%). Drainage limited to one segment was observed in 5.8% of patients. The median procedure time was 32.5 min (range, 9–144 min) (Table 2).

**TABLE 2:** Details of endoscopic ultrasound‐guided biliary drainage (EUS‐BD).

Drainage type, *n* (%)	Initial drainage	67 (47.5)
	Rescue drainage	74 (52.5)
Reasons for EUS‐BD	Inadequate or unsuccessful ERCP‐BD	74 (52.5)
	Intestinal obstruction	53 (37.6)
	Cholangitis	8 (5.7)
	SAA	6 (4.3)
Needles	19‐gauge	121 (85.8)
	22‐gauge	20 (14.2)
EUS‐BD stent configuration	HGS/HJS without additional EUS‐BD	120 (86.3)
	HGS plus bridging	8 (5.8)
	HGS plus AG	4 (2.9)
	HGS plus HDS	2 (1.4)
	AG alone	2 (1.4)
	AG plus bridging	1 (0.7)
	HDS alone	2 (1.4)
Drained number of liver segments by EUS‐BD	1	8 (5.8)
	2	48 (34.5)
	3	64 (46.0)
	4	10 (7.2)
	5	1 (0.7)
	7	8 (5.8)
Procedure time, min, median (range)		32.5 (9–144)

Abbreviations: AG, antegrade stenting; ERCP, endoscopic retrograde cholangiopancreatography; EUS‐BD, endoscopic ultrasound‐guided biliary drainage; HDS, hepaticoduodenostomy; HGS, hepaticogastrostomy; HJS, hepaticojejunostomy; SAA, surgically altered anatomy.

^a^ Stent configuration and the number of drained liver segments were evaluated in the 139 patients with technical success.

^b^ The stent configurations shown represent only the EUS‐BD component and do not include biliary stents placed by prior ERCP‐BD in patients undergoing rescue EUS‐BD.

### 3.3 Technical and Clinical Outcomes

The overall technical success rate of EUS‐BD was 98.5% (139/141). Two technical failures occurred: one due to the absence of IHBD dilation and one due to intraprocedural stent migration.

**FIGURE 2:**
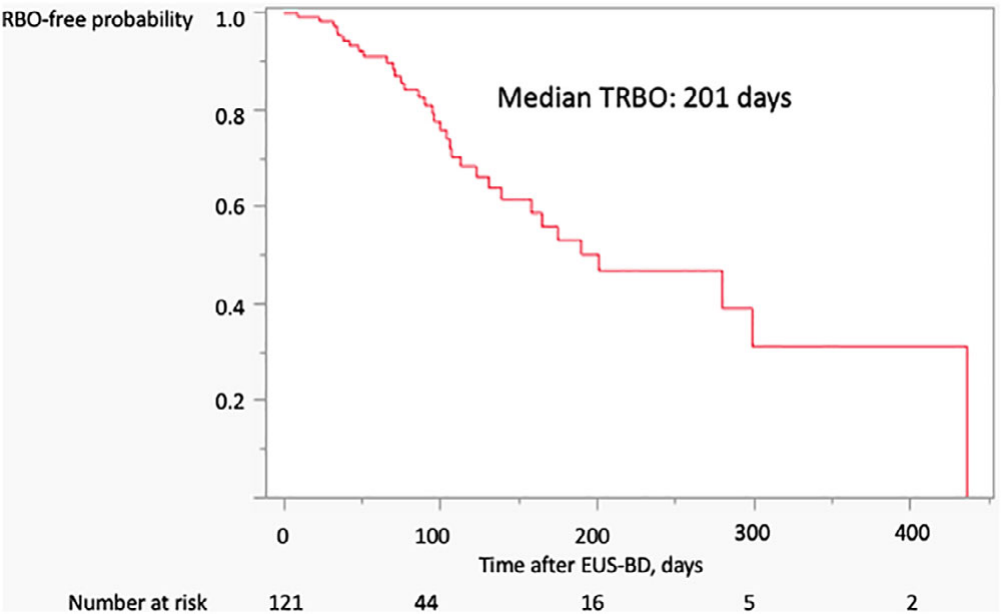
Kaplan–Meier curve for time to recurrent biliary obstruction after EUS‐BD. TRBO was evaluated in the 121 patients who achieved clinical success after technically successful EUS‐BD. The median TRBO was 201 days (95% CI, 139–436 days), and the median follow‐up period was 78 days (range, 6–436 days). EUS‐BD, endoscopic ultrasound‐guided biliary drainage; RBO, recurrent biliary obstruction; TRBO, time to recurrent biliary obstruction.

Among the 139 patients with technically successful procedures, clinical success was achieved in 121 patients, resulting in a clinical success rate of 87.1%. The primary reason for clinical failure was insufficient reduction in serum bilirubin levels (*n* = 17), while one patient failed to show symptomatic improvement despite technically successful drainage (Table 3). Among the 121 patients who achieved clinical success, RBO occurred in 34 patients. The median time to recurrent biliary obstruction was 201 days (95% CI, 139–436 days), and the median follow‐up period was 78 days (range, 6–436 days). The estimated RBO‐free probabilities are shown in Figure 2.

**TABLE 3:** Outcomes of endoscopic ultrasound‐guided biliary drainage (EUS‐BD).

Technical success, % (*n*)		98.5% (139/141)
Reasons for failure, *n*	No dilation of IHBD	1
	Stent migration	1
Clinical success, % (*n*)		87.1% (121/139)
Reasons for failure, *n*	Failed bilirubin reduction	17
	Failed symptom improvement	1
AE, % (*n*)		10.6% (15/141)
Details of AEs, *n*	Bile peritonitis	10
	Biloma	1
	Bleeding	1
	Cholangitis	1
	Liver abscess	1
	Stent migration	1

Abbreviations: AE, adverse event; EUS‐BD, endoscopic ultrasound‐guided biliary drainage; IHBD, intrahepatic bile duct.

### 3.4 Adverse Events

Procedure‐related AEs were observed in 15 patients, yielding an overall AE rate of 10.6%. The most frequent AE was bile peritonitis (*n* = 10). Other AEs included biloma, bleeding, cholangitis, liver abscess, and stent migration (one case each). All AEs were managed conservatively, except for a liver abscess, which required percutaneous transhepatic abscess drainage, and no procedure‐related mortality was reported (Table 3).

### 3.5 Factors Associated With Clinical Failure

Univariable and multivariable logistic regression analyses of factors associated with clinical failure are shown in Table 4. In the multivariable analysis, Bismuth type IV strictures and EUS‐BD drainage limited to one or two liver segments were significantly associated with clinical failure. Bismuth type IV strictures were significantly associated with higher odds of clinical failure (odds ratio [OR], 5.89; 95% CI, 1.72–24.3; *p* = 0.007). In addition, EUS‐BD drainage limited to one or two liver segments was also significantly associated with higher odds of clinical failure (OR, 5.11; 95% CI, 1.49–21.2; *p* = 0.014). Age (≥75 years), gender, and the use of EUS‐BD as rescue drainage were not significantly associated with clinical failure in the multivariable analysis.

**TABLE 4:** Univariable and multivariable analyses of factors associated with clinical failure.

	Clinical success, *n*		Univariable analysis			Multivariable analysis		
	Success	Failure	OR	95% CI	*p*‐Value	OR	95% CI	*p*‐Value
Age (≥75 years)	73	8	0.53	0.19–1.43	0.21	0.57	0.18–1.83	0.35
Men	70	8	0.58	0.22–1.58	0.29	0.39	0.11–1.26	0.12
Bismuth IV	35	14	8.60	2.65–27.95	<0.001	5.89	1.72–24.3	0.007
Rescue EUS‐BD	60	13	2.64	0.89–7.87	0.081	1.46	0.40–5.66	0.57
EUS‐BD of 1 or 2 segments	42	14	6.58	2.04–21.27	0.002	5.11	1.49–21.2	0.014

Abbreviations: CI, confidence interval; EUS‐BD, endoscopic ultrasound‐guided biliary drainage; OR, odds ratio.

^a^ The analysis was performed in 139 patients with technical success. Both technical failures occurred in patients with Bismuth type IV obstruction; therefore, 49 patients with Bismuth type IV obstruction were included in this analysis.

## 4 Discussion

This large multicenter study demonstrated that EUS‐BD is a highly feasible and relatively safe option for MHBO, achieving a high technical success rate of 98.5% and an AE rate of 10.6%. However, the clinical success rate was relatively modest at 87.1%, even when technical success was achieved. Bismuth type IV obstruction and EUS‐BD drainage limited to one or two liver segments were identified as factors associated with clinical failure.

EUS‐BD is now an established rescue drainage option for MDBO after failed ERCP‐BD, particularly in expert centers. Current ASGE and ESGE guidelines recommend EUS‐BD over PTBD for MDBO after failed ERCP‐BD when appropriate expertise is available [11, 12]. However, evidence regarding EUS‐BD for MHBO remains limited because of the complex biliary anatomy, heterogeneous drainage strategies, and narrower indications. Previous studies have mainly consisted of small retrospective series or case reports. Minaga et al. [13] evaluated EUS‐BD for MHBO as rescue drainage after failed transpapillary re‐intervention in 30 patients and reported technical and clinical success rates of 96.7% and 75.9%, respectively. Ogura et al. [14] reported corresponding rates of 100% and 90% in 10 patients. Our results were generally consistent with these previous reports. In the present study, the technical success rate was similarly high (98.5%; 139/141), whereas the clinical success rate was relatively modest (87.1%; 121/139), which is generally consistent with these previous reports.

Several technical approaches have been proposed to increase the drainage area in MHBO. EUS‐HGS is mainly used to access the left IHBD, whereas EUS‐HDS may provide access to the right IHBD. Bridging stenting from the left to the right biliary system, with or without AG, may also increase the drainage area. However, these complex approaches have been evaluated only in small case series [15, 16]. Ogura et al. [15] reported the technical feasibility of bridging stenting in 7 patients, whereas Moryoussef et al. [16] reported that bridging was feasible in only three of six patients. Given the need to traverse the obstruction and the steep angulation between the left and right bile ducts, the feasibility of bridging stenting in MHBO may be limited. In our cohort, bridging stenting was performed in nine patients, and clinical success was achieved in all of these cases; however, the small number of patients precludes any firm conclusion regarding its efficacy. Collectively, these data suggest that the technical feasibility of EUS‐BD for MHBO is high, although certain techniques—particularly bridging stenting—require further evaluation. The clinical success rate may be lower than that for MDBO because of limitations in the drained area. The main contribution of the present study is not the demonstration of superior technical or clinical success rates, but the analysis of one of the largest multicenter cohorts of EUS‐BD for MHBO, including both primary and rescue settings.

In the present study, nearly half of the patients underwent EUS‐BD as the primary drainage modality. This finding should be interpreted cautiously because primary EUS‐BD was not selected according to a standardized protocol. Instead, the drainage modality was determined by the treating endoscopist at each institution based on biliary anatomy, the expected difficulty of ERCP‐BD, gastrointestinal anatomy, the presence of intestinal obstruction or cholangitis, and the patient's general condition. Therefore, primary EUS‐BD in this study should not be interpreted as a universally recommended first‐line strategy for MHBO, but rather as a drainage option selected in expert centers for patients considered suitable for EUS‐BD.

Regarding factors associated with clinical failure, multivariable analysis identified Bismuth type IV obstruction and EUS‐BD drainage limited to one or two liver segments as factors associated with clinical failure. These findings suggest that the complexity of hilar obstruction and the drainage area achieved through EUS‐BD may influence clinical success. These findings should be interpreted cautiously, particularly because the total drainage area achieved by combined ERCP‐BD and EUS‐BD was not evaluated. Nevertheless, EUS‐BD for MHBO can be performed not only as a primary approach but also as a rescue technique after ERCP‐BD. In the rescue group, EUS‐BD was performed in addition to prior ERCP‐BD and therefore represented a combined drainage strategy. Although rescue versus primary EUS‐BD was not associated with clinical failure, the drainage area achieved by prior ERCP‐BD was not uniformly recorded. Importantly, the stent configurations shown in Figure 1 and Table 2 represent only the EUS‐BD component. Thus, the high proportion of HGS/HJS without additional EUS‐BD routes should not be interpreted as indicating that HGS/HJS alone was sufficient for overall BD in most patients with MHBO, because more than half of the patients underwent rescue EUS‐BD after prior ERCP‐BD. Therefore, the independent contribution of ERCP‐BD and EUS‐BD to the overall drainage effect could not be evaluated. Our cohort included multiple stent configurations (Figure 1). Selection of a configuration that achieves a larger drainage area may be important, although the technical feasibility of complex configurations, such as bridging stenting, requires further evaluation. Therefore, although these techniques may expand the drainage area achieved through EUS‐BD, further prospective studies are needed to determine the optimal EUS‐BD strategy for MHBO.

The AE rate of EUS‐BD for MHBO was 10.6% (15/141) in our study, and most AEs were mild, except for one moderate case requiring percutaneous abscess drainage. A large meta‐analysis including 155 studies (7887 patients) reported an overall AE rate of 13.7% (95% CI, 12.3%–15.0%) for EUS‐BD [17]. In addition, a review article reported a pooled AE rate of 8% (7/87) for EUS‐BD in MHBO [18]. Taken together with our results, the AE rate of EUS‐BD for MHBO appears comparable to that reported for EUS‐BD in general.

This study has several limitations. First, its retrospective multicenter design may have introduced selection bias, inter‐center variability, and temporal heterogeneity. Second, the indications for EUS‐BD and stent configuration were not standardized. In rescue cases after ERCP‐BD, the total drainage area achieved by combined ERCP‐BD and EUS‐BD could not be evaluated. Third, although TRBO was assessed, other clinically relevant outcomes, including chemotherapy initiation and overall survival, were not uniformly collected. Fourth, the number of drained liver segments was determined after EUS‐BD and should be interpreted as a procedural factor. Finally, because only 18 patients experienced clinical failure, the multivariable analysis may have been susceptible to overfitting and should therefore be interpreted as exploratory. These findings require validation in larger prospective studies.

In conclusion, EUS‐BD appears to be an effective and safe option for managing MHBO as both a primary drainage approach and a rescue approach after ERCP‐BD. In rescue cases, EUS‐BD should be interpreted as part of a combined drainage strategy with prior ERCP‐BD rather than as a standalone approach. Bismuth type IV strictures and EUS‐BD drainage limited to one or two liver segments were associated with clinical failure. Prospective, controlled studies are needed to establish optimal indications, techniques, and drainage strategies for EUS‐BD in MHBO.

## Author Contributions

**Takuji Iwashita** contributed to the study conception and design, data interpretation, drafting of the manuscript, and critical revision of the manuscript. **Kazuki Hayashi**, **Michihiro Yoshida**, **Reiko Yamada**, **Koichiro Matsuda**, **Shinichi Morita**, **Tadahisa Inoue**, **Kazuhiko Shioji**, **Keisuke Iwata**, **Akinori Maruta**, **Nobuhiko Hayashi**, **Masaaki Yano**, and **Ichiro Yasuda** contributed to data acquisition, data interpretation, and critical review of the manuscript. **Takuji Iwashita** and **Ichiro Yasuda** supervised the study.

## Funding

The authors have nothing to report.

## Ethics Statement

The study protocol was approved by the Institutional Review Board of Gifu University Hospital (GUH; approval number 2024‐019), which served as the central ethics committee covering all participating institutions.

## Consent

N/A

## Conflicts of Interest

The authors declare no conflicts of interest.

## Data Availability

The data that support the findings of this study are available on request from the corresponding author. The data are not publicly available due to privacy or ethical restrictions.
